# DNA in 3R: Repair, Replication, and Recombination

**DOI:** 10.1155/2012/658579

**Published:** 2012-03-28

**Authors:** Frédéric Coin, Bernardo Reina-San-Martin, Giuseppina Giglia-Mari, Mark Berneburg

**Affiliations:** ^1^IGBMC, 67404 Illkirch Cedex, France; ^2^IPBS, 31077 Toulouse, France; ^3^University of Tuebingen, Germany

This special issue entitled “DNA in 3R: Repair, Replication, and Recombination” is dedicated to biological processes that preserve the integrity of our genome. These phenomena have attracted broad interest among a large community of scientists that cross disciplines from mathematics, physics, chemistry, and biology to clinical scientists. DNA is continuously exposed to a range of damaging agents, including reactive cellular metabolites, environmental chemicals, ionizing radiation, and UV light. The biochemical consequences of DNA lesions are diverse and range from obstruction of fundamental cellular pathways like transcription and replication to fixation of mutations. Cellular misfunctioning, cell death, aging, and cancer are the phenotypical consequences of DNA damages accumulation in the genome. Fortunately, an intricate set of genome surveillance mechanisms function to counteract genomic insults. Among these mechanisms, base excision repair and nucleotide excision repair are both dedicated to the removal of single-strand lesions contrary to double-strand break repair. Additionally, some specialized polymerases can temporarily take over lesion-arrested DNA polymerases during S-phase, in a mutagenic mechanism called translesion synthesis. Such polymerases only work if a more reliable system, such as homologous recombination, cannot avoid stumbled DNA replication. These DNA repair mechanisms function in conjunction with an intricate machinery of damage sensors, responsible of a series of phosphorylations and chromatin modifications that signal to the rest of the cell the presence of lesions on the DNA. Together DNA repair mechanisms and DNA damage signaling systems form a molecular shield against genomic instability called DNA Damage Response system. Hence, we have tried to integrate several papers that present a synergy that emerge when researchers from different fields put their forces together into a common goal, trying to improve human health. We thank the contributors for their work and the many reviewers who served conscientiously and tirelessly to assure an issue that meets the standards.

*Frédéric Coin* *Frédéric Coin* *Bernardo Reina-San-Martin* *Bernardo Reina-San-Martin* *Giuseppina Giglia-Mari* *Giuseppina Giglia-Mari* *Mark Berneburg* *Mark Berneburg*

